# The Role of Exosomes in Epithelial–to-Mesenchymal Transition and Cell Functional Properties in Head and Neck Cancer

**DOI:** 10.3390/cancers15072156

**Published:** 2023-04-05

**Authors:** Nicholas S. Mastronikolis, Efthymios Kyrodimos, Despoina Spyropoulou, Alexander Delides, Evangelos Giotakis, Zoi Piperigkou, Nikos K. Karamanos

**Affiliations:** 1 Department of Otorhinolaryngology—Head and Neck Surgery, School of Medicine, University of Patras, 26504 Rion, Greece; 2 1st Otolaryngology Department, School of Medicine, National & Kapodistrian University of Athens, ‘Ippokrateion’ General Hospital, 11527 Athens, Greece; 3 Department of Radiation Oncology, Medical School, University of Patras, 26504 Rion, Greece; 4 2nd Otolaryngology Department, School of Medicine, National & Kapodistrian University of Athens, ‘Attikon’ University Hospital, 12462 Athens, Greece; 5 Biochemistry, Biochemical Analysis & Matrix Pathobiology Research Group, Laboratory of Biochemistry, Department of Chemistry, University of Patras, 26504 Patras, Greece; 6 Foundation for Research and Technology-Hellas (FORTH), Institute of Chemical Engineering Sciences (ICE-HT), 26504 Patras, Greece

**Keywords:** exosomes, head and neck cancer, epithelial-to-mesenchymal transition, metastasis, drug resistance, extracellular matrix

## Abstract

**Simple Summary:**

According to WHO 2018 estimates, cancer is the second leading cause of mortality worldwide after cardiovascular disease, accounting for one in six fatalities. Mortality due to head and neck cancer is also significantly high, making it the seventh most prevalent cancer. Elucidating the mechanisms of action of key regulators, such as bioactive molecules secreted by the cancer cells in exosomes, their interaction with neighboring and distant cells, the extracellular matrix, and the tumor microenvironment, could be a valuable tool for future diagnostic and treatment approaches. Exosomes facilitate several critical functions in cancer cells, such as epithelial-to-mesenchymal transition, which makes head and neck cancer even worse by giving it a metastatic potential to evade the secondary site and spread cancer in the body. Exploring the molecules involved in this process could help targeting specific sites, for instance, by modifying the cell-specific proteins and exosome cargoes.

**Abstract:**

Exosomes are nanosized vesicles that are produced in normal and cancer cells, promoting intracellular communication. In head and neck cancer (HNC), exosomes are involved in many undesirable events of cancer development and progression, including angiogenesis, tumor microenvironment (TME) remodeling, invasion, epithelial-to-mesenchymal transition (EMT), metastasis, extracellular matrix (ECM) degradation, and drug resistance. Exosomes are involved in altering the signaling pathways in recipient cells by the cargoes they carry. Proteins, lipids, and nucleic acids such as DNA fragments and RNAs (i.e., mRNAs, miRNAs, and long non-coding RNAs) are carried in the exosomes to promote cell communication. EMT is a critical cellular process in which epithelial cells are forced to become mesenchymal cells by the actions of SNAIL/SLUG, TWIST, and ZEB family transcription factors carried in exosomes that facilitate metastasis. In this critical review, we focused on exosome biogenesis, their cargoes, and their involvement in EMT induction and metastasis during HNC. Insights into exosome isolation and characterization, as well as their key role in ECM remodeling and degradation, are also presented and critically discussed. More importantly, this article addresses the role of exosomes in HNC and drug resistance induced in drug-sensitive cancer cells. In addition, exosomes have a great potential to be used as diagnostic and therapeutic tools. A better understanding on exosome biogenesis, composition, and functions in HNC will aid in developing novel therapeutic strategies to treat HNC, overcome therapy resistance, and avoid metastasis, which is a significant cause of cancer death.

## 1. Introduction

Exosomes are extracellular double-layered membranous vesicles with sizes ranging from 30 to 150 nm. The term “exosome” was first introduced by Trams et al. in 1981 for the alternate membrane fragments recovered from body fluids and later coined by Rose Johnstone for extracellular vesicles (EVs) jettisoned from maturing blood reticulocytes [1,2]. These secretory vesicles are endosome-derived and released into the blood, urine, saliva, cerebrospinal fluid (CSF), etc., from various mammalian cells. Exosome biogenesis begins with endocytosis to form early endosomes that mature further to become late endosomes. Inward budding results in multivesicular endosome (MVE) formation and intraluminal endosomal vesicles (ILVs) which are released into extracellular space upon fusion with the cell membrane. Exosomes transport a range of functional proteins/glycoproteins, including proteoglycans/glycans, and enzymes, but also genetic cargoes, such as DNA and RNA (i.e., mRNAs, miRNAs, and long non-coding RNAs) from healthy and malignant cells [3,4]. The EVs secreted outside cells are highly heterogenous, including exosomes and other membranous structures, such as microparticles, macrovesicles, oncosomes, and apoptotic bodies. Each EV differs from others in terms of origin, size, and various molecules loaded in it.

Hence, tracking exosomes is considered an easy process in serious pathological conditions such as cancer, a leading cause of death worldwide. Cancer cells are highly active and therefore produce exosomes in the TME at a higher rate [5]. Therefore, exosomes may act as an alternate non-invasive diagnostic or prognostic marker and treatment tool for cancers such as head and neck cancer (HNC), making treating it less challenging with surgery, systemic therapy, and radiotherapy. HNC is traditionally considered a disease linked to alcohol and tobacco abuse and oncogenic viruses such as human papillomavirus (HPV) and Epstein-Barr virus (EBV) [6]. It was responsible for 450,000 HNC-related mortalities in 2018 and is the seventh most common type of cancer worldwide [6,7]. It affects the upper aerodigestive tract, excluding the brain, ear, eye, and esophagus. It includes cancers of the oral cavity, pharynx (naso-, oro-, and hypopharynx), larynx, thyroid, salivary glands, and other maxillofacial tumors, making it a diverse group. Predominantly, squamous cell carcinoma (SCC) accounts for over 90% of head and neck cancers [8]. Tumor-derived exosomes in TME are considered an excellent tool for exchanging bioactive substances between tumor cells and the TME for cell–cell communication. These exosomes carrying the bioactive substance (i.e., signaling molecules and metabolites) are responsible for vascular permeabilization, angiogenesis, fibroblast activation, inflammation, mediating intercellular communication, and EMT, promoting the metastatic behavior in the cells of the head and neck [9,10,11,12,13]. During EMT, cell morphology reversibly transforms into the mesenchymal state from the epithelial state. It is well established that E-cadherin expression in epithelial cells is downregulated, vimentin is upregulated, and β-catenin is relocalized from the membrane to the nucleus, resulting in decreased adhesion, loose connection with the ECM, a loss of apical polarity, and basal anchoring [14,15]. It is a crucial process before exosome recipient head and neck epithelial tumor cells attain migratory and invading properties. HNC covers numerous subsites, and with unique characteristics, they regulate EMT through distinct and overlapping mechanisms.

In this article, we summarize the role of exosomes in EMT and critical cell properties in the case of specific HNCs. The effects of different exosome contents on tumor cell metastatic potential, TME, drug resistance, biogenesis, isolation, and characterization, and their use in diagnosis and therapeutic interventions are also presented and critically discussed.

## 2. Biogenesis, Structural and Functional Features of Exosomes

### 2.1. Exosomes: Biogenesis, Cargo Composition, Isolation, and Characterization

**Figure 1:**
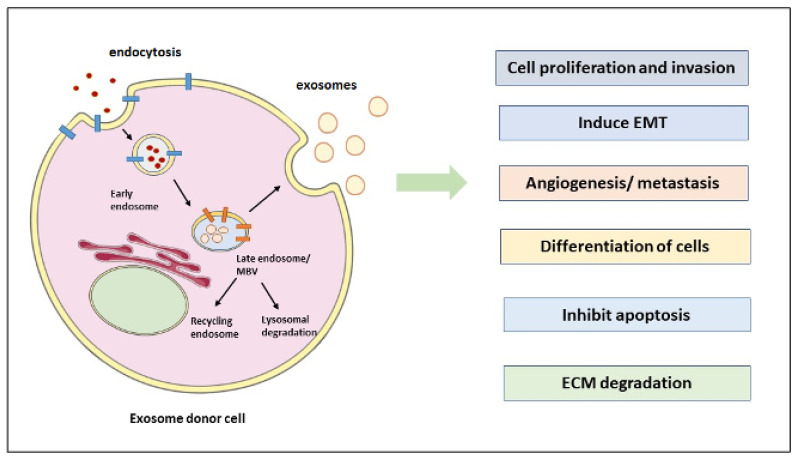
Exosome biogenesis and its role in the tumor microenvironment. Extracellular components (depicted in red) are endocytosed and packed into early endosomes following maturation in late-sorting endosomes to become MVBs. Cancer cells release exosomes that are correlated with tumor microenvironment and tumor progression. Cancer-derived exosomes release their cargoes (i.e., lipids, proteins, enzymes, metabolic products, DNA/RNA) that, apart from the key roles in cancer cell properties, form pre-metastatic niches and stimulate metastatic tumor potential. Blue and orange lines depict proteins involved in endocytosis and multivesicular specific proteins. Abbreviations: ECM, extracellular matrix; EMT, epithelial-to-mesenchymal transition; MVB, multivesicular bodies.

The understanding of EVs has improved dramatically and has reached beyond just a “platelet dust” definition that was given early in the 1960s [16]. A crucial shift from knowing them as garbage carriers to a new mode of intercellular communication has made their role essential in clinical applications. Exosomes were discovered in 1946 by the experiments conducted by Chargaff and West on human plasma [17]. After determining that plasma clotting is inhibited by removing the pelleted plasma fraction following high-speed centrifugation, Peter Wolf later found that these clotting suppressors are 20–50 nm vesicles produced from platelets [17]. Research papers in Journal of Cell Biology (JCB) and Cell published in 1983 also supported the existence of exosomes by showing 50 nm active vesicles interacting with transferrin receptors on reticulocytes derived from maturing reticulocytes in sheep and secreted into the extracellular space [18]. Exosome biogenesis is an intensely regulated process that gets activated by cell-specific receptors and the downstream signaling pathways [19]. Exosomes can arise from exosomes budding into discrete endosomes. They mature into multivesicular bodies (MVBs) and are released after MVB fusion with the plasma membrane of the mammalian cells, as shown in Figure 1. Exosome biogenesis can also arise by direct vesicle budding from the plasma membrane as an immediate process and budding at intracellular plasma membrane-connected compartments (IPMCs) followed by a deconstruction of IPMC neck(s), which is a delayed process [20].

The formation of exosomes starts with the endocytosis of extracellular components in early endosomes (EEs) and then successive maturation into late-sorting endosomes [19,20]. They finally become MVBs, containing several ILVs by inward membrane budding, sequestering cargoes such as proteins, proteolytic/glycolytic enzymes, metabolic products, DNA, and RNA [21].

These vesicles are also degraded by fusion with lysosomes or recovered through the back-fusion pathway from the plasma membrane [22]. The released exosomes from donor cells deliver cargo to recipient cells through endocytosis, fusion with the plasma membrane, and receptor–ligand interactions. Notably, protein classification in endosomes is crucial for endosome maturation, and the endosomal sorting complex required for transport (ESCRT) mechanism is an essential contributor to this activity. ESCRT machinery in the protein sorting of ILVs is a highly controlled mechanism; however, protein sorting of ILVs can sometimes be an ESCRT-independent event. ESCRT apparatus consists of ESCRT-0, -I, -II, and -III. ESCRT-0 and ESCRT-I recruit -II and cargoes, followed by ESCRT-II recruitment of -III to assist with ILV budding. In the ubiquitin-dependent pathway, ESCRT-0 recognizes mono-ubiquitinated proteins assisted by signal-transducing adaptor molecules (STAM1/2) and hepatocyte growth factor (HRS) heterodimer [23,24,25]. HRS-recruited-clathrin helps to encounter the ubiquitinated cargo. This event is further followed by the joining of ESCRT-0 to -I and -II with a higher affinity to ubiquitinated substrates on the endosomal membrane. From here, it undergoes inward budding, and the process of ILV formation ends with pinching off the membrane by joining ESCRT-III to the complex and releasing the buds into the endosome [26]. ILVs are secreted into extracellular space by cell membrane fusion of MVBs once de-ubiquitinated. If de-ubiquitylating enzymes (DUBs) do not act, ILVs are targeted to lysosomes for degradation [27]. ATPase vacuolar protein sorting-associated protein 4 (VPS4), with its co-factor vesicle trafficking (VTA), dissociates the ESCRT complex at the end of exosome biogenesis [23]. Alix protein interacts with ESCRT-III and supports the intraluminal budding of endosomal membranes. This protein helps deliver un-ubiquitinated cargoes such as proteinase-activated receptor 1 (PAR1), directly interacting with it or carrying the syndecans and tetraspanin CD63 indirectly. Syntenin (syndecan adaptor) interacts directly with Alix through LYPX(n)L motifs [28].

ESCRT-independent mechanisms such as RAS-related protein in brain 31 (RAB31) and tetraspanin pathway take place in lysosome/endosome-related organelle called melanosomes present in melanocytes. Premelanosome protein 17 (PMEL17) and tetraspanin CD63 mediate the melanosome membrane invagination to contribute to ILV formation [29,30]. Exosome secretion takes place by membrane fusion, which is assisted by soluble N-ethyl maleimide (NEM)-sensitive factor attachment protein receptor (SNARE) complex. Rab27A and Rab27B induce MVBs transfer to the cell membrane and mediate exosome release [31].

Exosome composition and content reflect their site of origin or donor cells. These exosomes contain many active molecules such as lipids, transcription factors, receptors, ECM proteins/glycoproteins, enzymes, and nucleic acids (i.e., DNA, mRNA, ncRNA) inside the lumen and on the membranous surface. These molecules are decorated on vesicles that can be cell- or tissue-specific and can be familiar to all the exosomes. For instance, reticulocyte-specific types of exosome proteins include tetraspanins, cell adhesion molecules (CAMs), major histocompatibility complex (MHC) class I and II, integrins, and transferrin receptors (TfR), and protein common to all include heat shock proteins (i.e., Hsc70 and Hsc90), fusion and transferring proteins (i.e., annexin, flotillin, Rab2, and Rab7), cytoskeleton proteins (i.e., actin, myosin, tubulin), and proteins such as tetraspanins (i.e., CD9, CD63, CD81, and CD82), Alix that mediate MVBs formation. Lipids such as lyosbisphosphatidic acid (LBPA) in exosomes facilitate the inward budding of ILVs in MVBs. Sphingomyelin, phosphatidylcholine, and BMP help distinguish the EVs, sphingomyelin being high in exosomes. The fusion of these exosomes is responsible for the change in the lipid composition of the recipient cells [32]. Apart from proteins and lipids, nucleic acids, such as dsDNA [phosphatase and TENsin (PTEN), MutL homolog 1 (MLH1), and tumor protein 53 (TP53), non-coding RNAs (ncRNAs)], which include long ncRNAs (lncRNAs), microRNAs (miRNAs) and circular RNAs (circRNAs), are also present in exosomes [33]. It is worth noticing that microRNAs post-transcriptionally regulate gene expression, affecting cell proliferation and cell death [34]. LncRNAs are longer than miRNAs (20–24 nucleotides long) and are regulatory and non-coding molecules. LncRNA dysregulation leads to cancer progression through epigenetic, transcriptional, and post-transcriptional mechanisms. TCF21 antisense RNA inducing demethylation (TARID), antisense 1 dehydrogenase/reductase 4 (AS1DHRS4), and potassium voltage-gated channel subfamily Q member 1 (Kcnq1ot1) are some exosomal lncRNAs that recruit DNA methyltransferases to change the chromatin conformation [35,36]. miRNAs are primarily present in the blood and saliva in exosomes, and these miRNAs can mediate transcriptional regulation, mRNA degradation, and translational suppression [37]. circRNAs are the covalently closed continuous loops of RNA that regulate gene expression and are associated with HNCs [38].

The message here could be summarized as follows: exosome composition and content are altered when they originate from a cancer cell [39]. These tumor-derived exosomes (TDEs) act as tools for interchanging substances at short and long distances with tumor cells, the TME, and nearby healthy cells [40]. Intercellular communication by TDEs promotes cancer cell proliferation, EMT generation, angiogenesis, and pre-metastatic niche (PMN) formation [40].

Isolation and characterization of exosomes are critical procedures for functional studies at the cell level as well as at the diagnostic one. The assessment of the tumor profile is made using tissue biopsies. However, taking tissue samples using needles or surgery is not only highly invasive but also anatomical; the location of the tumor makes accessibility poor. Exosomes secreted by tumors, on the other hand, are highly accessible due to their presence in body fluids such as blood, saliva, urine, CSF, etc. Liquid biopsy has a lot of advantages over tissue biopsy, some of which are non-invasiveness, real-time monitoring facility, and early diagnosis of cancers. However, circulating tumor DNA and CTC require large amounts of blood, fresh samples, and quick sample processing, while exosomes in body fluid samples are easy to handle. They can be frozen, present in higher concentrations, and are present earlier in metastatic disease than CTCs [41].

For exosome isolation from biological fluid and cell culture supernatants, various isolation procedures are used, such as polyethylene glycol-mediated precipitation, ultracentrifugation, immunoaffinity capture, and microfluidics [5]. Due to greater vesicle recovery and uniformity, as well as a decreased danger of complex protein aggregation and vesicle loss due to ultracentrifugation, the mini-SEC method is the most advantageous approach among all others. The characterization of exosomes is commonly performed by electron microscopy (EM), nanoparticle tracking analysis, tuneable resistive pulse sensing, immunodetection methods (i.e., Western blotting, flow cytometry), and mass spectrometry (MS).

Exosome identification and verification are assessed using electron microscopy by direct exosome visualization [42]. However, this technique cannot be used on a daily basis. A consistent loss of exosomes is observed during dehydration and embedding procedures [42]. To obtain the size concentration and distribution of exosomes nanoparticle, tracking analysis is used. By using laser light dispersion, this technique ranks and sizes the nanoparticles suspended in a liquid using an integrated analysis of Brownian motion [43]. Tunable resistive pulse sensing determines the amount and size of particles present in the exosome. In immunodetection methods, exosome cargoes specifically bind to the mono- or polyclonal antibodies. Western blotting is one such method. This method is also used as a semi-quantitative densitometry analysis of protein bands that gives insight into disease progression. Flow cytometry also quantitively analyses exosome cargoes using streptavidin-coated magnetic beads and biotin-labeled anti-CD63 Ab for a CD63+ exosome. For proteome characterization of exosomes, MS is one of the most favorable techniques. In this method, analytes are transformed into gaseous ions, and they are then characterized according to their mass-to-charge ratio. It provides sensitivity specificity, high throughput, and cost-effective testing. To measure histone alterations, transcription factors, and other chromatin-associated proteins, MS has also been coupled with chromatin immunoprecipitation.

### 2.2. Exosomes: Specificity and Sensitivity

The composition and structure of exosomes in the contest of the donor cell type also affect their specificity and selectivity. Indeed, the specificity/selectivity between the donor and recipient cells is an emerging research area. Despite many potential uses for exosomes, their selective trafficking and communication pathways remain unclear. As a matter of fact, selective fingerprinting of exosomes continues to be a controversial topic [44]. There are several proteins and lipids on the donor cell-derived exosomal membrane that interact with receptors on the surface of recipient cells. The uptake and signaling properties of exosomes are influenced by these interactions. Furthermore, the specific cargo profile of exosomes, which varies depending on the donor cell, affects its specificity/selectivity. A cargo can interact with specific receptors or pathways in the recipient cell, which determines the exosome’s functional effects. Mulcahy et al. examined the molecular mechanisms underlying the selective uptake of exosomes by recipient cells. They found that an exosomal tetraspanin CD63, which interacts with integrins on recipient cells’ surfaces, leads to exosome internalization [45]. It is noticeable that different donor cells produce exosomes with different protein and RNA profiles, which make them more specific to the recipient [46]. Thus, dendritic cell-derived exosomes selectively target T cells through the transfer of miR-155 [47], while mesenchymal stem cell-derived exosomes target cancer cells selectively through the transfer of miR-146b [48]. In another study, researchers found that exosomes derived from HNSCC cells inhibited natural killer cells’ activity by transferring programmed death-ligand 1 (PD-L1). They conclude that PD-L1+ exosomes circulating in HNSCC patients can be useful as markers of disease activity and immune evasion [49].

The concept of exosomes’ specificity/selectivity between donor and recipient cells is also important in understanding the uses of exosomes as a therapeutic tool. In a study by Sancho-Albero et al. [50], using hollow gold nanoparticles (HGNs) in exosome biogenesis, researchers followed cell–cell communication and clarified how the cellular origin of exosomes affects HGN transference between cells. Moreover, they investigated how to selectively induce death by hyperthermia only in specific cells using this preferential transfer. Their results indicate that the exosomal envelope provides a fingerprint that is responsible for transfer selectivity, which maintains the identity and compatibility of transference. Furthermore, they demonstrated that this preferential uptake can be utilized to selectively induce cell death by light-induced hyperthermia only in cells of the same type as those creating the loaded exosomes, thus enabling a better understanding of designing exosome-based selective therapies. In a recent review, He et al. [51] stated that due to their surface proteins and other features, exosomes are capable of carrying drugs specifically to target cells or tissues and can therefore be used as natural drug carriers in immunotherapy. Thus, exosomes can enhance therapeutic efficiency and improve drug pharmacokinetics, such as solubility, stability, and bioavailability, reducing side effects associated with non-specific distribution. Using surface modification technology, exosomal targeting optimization can be accomplished by improving the exosome’s enrichment ability in the specific part and its affinity to the target tissue. The technology could provide new ideas and strategies to target exosomes in clinical settings.

Overall, exosome specificity and selectivity for targeting recipient cells are complex processes dependent on the composition of the exosome membrane. A deeper understanding of the molecular mechanisms of these processes will enable the development of new therapies based on exosomes.

## 3. Exosomes in EMT, Functional Cell Properties, and Biological Procedures

**Figure 2:**
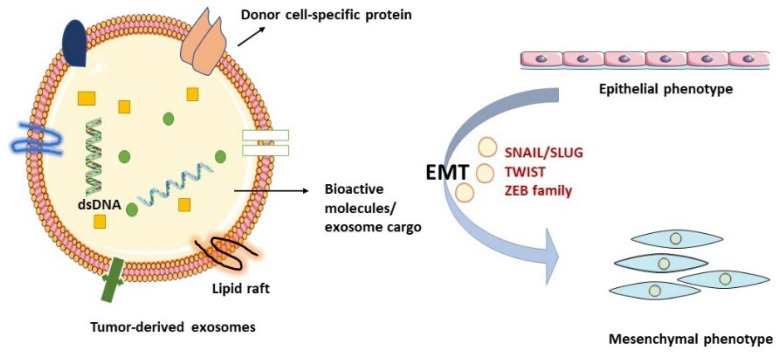
Schematic representation of the roles of tumor-derived exosomes in mediating EMT. The provisional matrix formed in cancer cells reprograms the primary cancer cells and evokes EMT that transforms the epithelial-like cells into mesenchymal-like cells through the major EMT transcription factors (i.e., SNAIL/SLUG, TWIST1, and ZEB). The dynamic interplay among cancer cells with the surrounding stroma through the cancer-derived exosomes governs the establishment of a pre-metastatic niche to globally mediate the multistep process of metastatic potential. Abbreviations: EMT, epithelial-to-mesenchymal transition; SNAL/SLUG, Zinc finger protein1/2; TWIST1, Twist Family BHLH Transcription Factor 1; ZEB, Zinc finger E-box-binding homeobox.

Metastasis is the spreading of tumor cells from the site of origin to distant part/s of the body. The process of metastasis could start with EMT. It is defined as a morphological change from the epithelial to the mesenchymal phenotype, and it comes with tumor occurrence, invasion, metastasis, and resistance to cancer therapies [52] (Figure 2). The ability to migrate through blood or lymphatic routes comes from EMT-associated proteins and molecules [53].

Several tumor-derived molecules, such as transforming growth factor (TGFβ), fibroblast growth factor (FGF), epidermal growth factor (EGF), hedgehogs, Wnt ligands, and interleukin-6 (IL-6), act as EMT inducers. These molecules initiate the downstream signaling TGFβ/SMAD, PI3K/protein kinase B (AKT), Wnt/β-catenin, and mitogen-activated protein kinases (MAPKs) signaling pathways. EMT-related gene expression is governed by the downstream transcription factors such as Zinc finger protein SNAI1 (SNAIL)/Zinc Finger Protein SNAI2 (SLUG), β-catenin, Twist-related protein 1 (TWIST), and Zinc finger E-box-binding homeobox 1/2 (ZEB1/2) [54]. Although EMT is a reversible process, the epithelial, mesenchymal, and a hybrid state (E/M cells) do exist with a high expression of E-cadherin in epithelial cells, N-cadherin and vimentin in mesenchymal cells, and E-cadherin, N-cadherin, and vimentin in the E/M cells [55]. EMT is a critical process in cancer stem cell generation (CSCs). EMT-inducing signals stimulate non-CSCs to become CSCs by activating the cell stemness markers such as CD133 and CD44 [56]. Exosomal proteins such as the SNAIL superfamily is often carried between cells in exosomes. In one study, exosomes isolated from TGFβ1-treated papillary thyroid carcinoma (PTC) cell line were found to induce EMT in naive PTC cells. LncRNA MALAT1 and EMT effectors SLUG and SOX2 were also observed to be dumped in normal thyroid cell lines. Along with exosomal linc-ROR, EMT induction was now seen in normal thyroid cell lines, which did not appear before [40]. Apart from the SNAIL protein superfamily, other transcription factors, such as matrix metalloproteinase-13 (MMP-13), are carried in hypoxic exosomes in nasopharyngeal carcinoma (NPC) cells. It induces metastasis in recipient cells through a hypoxia-induced factor-1α (HIF-1α)-dependent manner. Vimentin is upregulated, and the expression of E-cadherin is reduced in recipient cells [57]. Expression of exosomal miRNAs in some cases is negatively correlated with the degree of EMT. For example, overexpression of miR-34a-5p in cancer-associated fibroblast (CAF)-derived exosomes targets AXL (a tyrosine kinase receptor taking its name from the Greek word anexelekto, which means uncontrolled), reducing the activity of β-catenin, that in turn inhibits EMT in oral squamous cell cancer (OSCC) cells. Similarly, reduced expression of miR-34a-5p in CAF-derived exosomes can be transferred to OSCC cells by exosomes from fibroblasts. Exosomal cirRNAs compete with endogenous RNAs (ceRNAs) by capturing miRNAs in the cancer cells. For example, in PTC, miR-653-5p activity is inhibited by exosomal circ007293 by trapping it. This action of circ007293 increases the expression of paired box 6 (PAX6) in PTC cells and promotes the EMT of tumor cells [58].

The enhancement of metastatic potential also results from ECM degradation and turnover. ECM mainly consists of elastin, fibronectin, collagens, glycoproteins, glycosaminoglycans (i.e., hyaluronan, etc.), proteoglycans, and matricellular proteins [53,59,60]. Matrix macromolecules create a 3D dynamic meshwork, which is one of the most significant and abundant biomaterials in living organisms [61]. These complex macromolecular networks communicate through the cell receptors, and this dynamic interplay may lead to programmed cell death in normal cells when detached from ECM [53]. Both ECM stiffness, in a TGF-evoked mechanism, and ECM breakdown as an altered remodeling of provisional matrix [62] in an MMP-related pathway can contribute to cancer cell invasion by forming a bridge in the basement membrane and a path in the TME, respectively [63], reinforcing the establishment of the PMN. ECM enzymatic breakdown is achieved by MMPs, plasminogen activators, cathepsins, heparanase, and hyaluronidases that affect the EMT process and subsequently regulating invasion, motility, autophagy, exosome formation, angiogenesis, and ECM turnover [64]. A study demonstrated that exosome, rich in ECM degradation enzyme, transferred from MDA-MB-231 highly metastatic breast cancer cells to MCF-10A epithelial breast cancer cells (non-tumorigenic), leads to metastasis by increasing the expression of MMP-2 and MMP-9 [65,66]. ECM proteins periostin and fibronectin1 (FN1), induced during EMT, alter the composition of the ECM [67]. Periostin secreted outside cells acts in ECM organization and plays a role in cell adhesion. It interacts with integrins αvβ3 and αvβ5 and thereby stimulates cell adhesion. It also enables mobility through the FAK and Akt/PKB-mediated signaling pathways [68,69]. Overexpression of this protein is seen in many cancers, such as head and neck, gastric, colon, pancreatic, and thyroid malignancies [70,71,72,73,74]. Intracellular proteoglycan, named serglycin, is also secreted in the ECM and has a role in oncogenic signaling. It interacts with cell surface receptors such as CD44 and integrins and makes TME more pro-inflammatory and pro-angiogenic. This is achieved by the induction of IL-8, TGFβ2, VEGF, HGF, and CCL2 [75]. S100 proteins, a calcium-binding family, regulate calcium homeostasis, protein phosphorylation, and enzyme activity and act as transcriptional factors and organizations of cytoskeletal components. When upregulated, this protein degrades ECM, disrupts cell–cell adhesion and promotes metastasis [76,77]. As the crosstalk between cancer cell stroma and cell–cell is crucial for their invasion, they communicate intercellularly using exosomes and tunneling nanotubes (TNTs) [78]. The cancer cells can directly communicate with different neighboring cells using TNTs. Exosome released by cancer cells in ΤΜΕ interacts with ECM macromolecules to degrade or remodel them, and ECM takes care of the secretion and uptake of exosomes [21]. One example of ECM assisting in exosome uptake comes from a study where endothelial cells were shown to uptake the pancreatic adenocarcinoma-derived exosomes with enriched VCAM-1 and integrin α4 [79].

Metabolic reprogramming is a hallmark of cancer and occurs when cancer cells alter metabolic pathways to develop further. Recently, exosomes have emerged as important mediators of TME and metabolic reprogramming in recipient cells, thus contributing to cancer evolution and drug resistance [80]. By targeting main metabolic enzymes, exosomes can deliver metabolites and signal molecules to recipient cells, such as proteins, lipids, and nucleic acids, altering their metabolic state [81]. One example of how exosomes contribute to metabolic reprogramming is the regulation of glucose metabolism. Cancer cells are often characterized by increased glucose uptake and use, a process known as the Warburg effect. It has been demonstrated that exosomes secreted by cancer cells increase glucose uptake and use by transferring glucose transporter proteins, such as GLUT1 [82]. Another example of exosome implication in tumor metabolic reprogramming is their involvement in the regulation of lipids metabolism. During cancer progression, tumor cells require an increased number of fatty acids to create their membranes and produce energy. Thus, cancer cells secrete exosomes that transfer lipids and lipoproteins to recipient cells, improving fatty acid synthesis [83]. Additionally, exosomes can promote tumor immune evasion by regulating the metabolism of immune cells in the tumor microenvironment [80]. In conclusion, a better understanding of how exosomes mediate these processes may lead to new therapeutic targets for cancer treatment.

Circadian rhythm is the internal biological clock that regulates various physiological functions such as sleep–wake cycles, metabolism, and gene expression in living organisms. It has also been demonstrated that it can regulate exosome secretion in various cell types. Using a mice model of chronic nocturnal shift work, Khalyfa et al. found that gut microbiota changes and colonic cell permeability alter plasma exosome cargo and function, possibly by causing systemic inflammation and altering circadian clock expression in target tissues [84]. Several pathologies, including malignancies, are influenced by the circadian rhythm. There is evidence that altered circadian clock gene expression is associated with several cancers, but little is known about abnormal circadian clocks in head and neck squamous cell carcinomas (HNSCCs). Disruption of circadian clock gene expression was found to be associated with the progress of HNSCC by Hsu et al. [85]. An analysis of nine circadian clock genes was conducted on 40 HNSCC patients and their surrounding healthy tissue. Researchers found that the patients had significantly lower expression levels of PER, CRY1, and BMAL1 genes and were especially downregulated in the advanced stages of the disease. In this study, however, it was unclear whether exosomes play a role in this process. In summary, circadian rhythms regulate exosome secretion, and exosomes may be involved in mediating the effects of chronic circadian rhythm disruption on metabolic health. Therefore, exosomes could be used as potential markers to monitor circadian rhythm and detect early warning signals of rhythm dysfunction. Moreover, exosomes may be used therapeutically to regulate and maintain the internal circadian rhythm system and to prevent or treat diseases caused by its dysfunction [86]. However, to fully understand this relationship, further research is needed.

## 4. Exosomes and HNC

**Figure 3:**
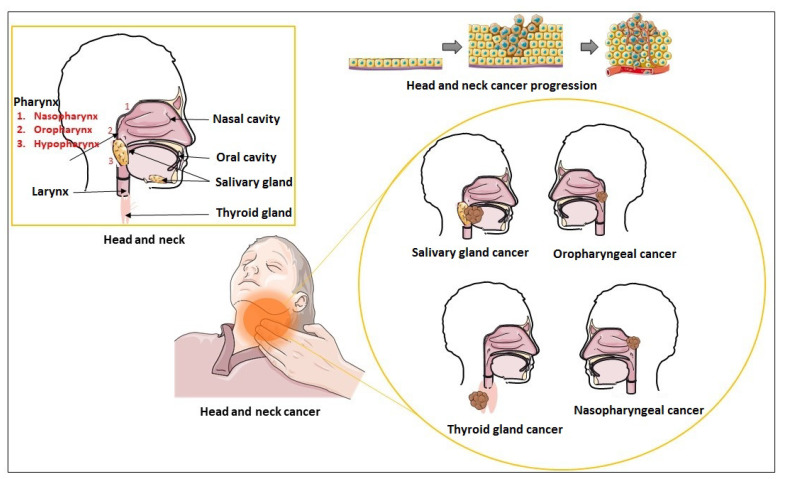
Schematic representation of head and neck region and types of head and neck carcinoma. The epithelial cell lining in the head and neck begins to proliferate and become metastatic later, which metastasizes to other parts of the head, neck, or other body parts. This type of cancer occurs in the upper shared respiratory/digestive tract due to alcohol abuse, smoking, and viruses such as human papillomavirus.

HNC is a collection of epithelial malignancies that begins primarily in the squamous cells lining the mucosal surfaces. These cancers are called head and neck squamous cell carcinomas and are usually metastasized in the head and neck lymph nodes. The upper shared respiratory/digestive tract includes lips, oral cavity, oropharynx, nasal cavity, nasopharynx, hypopharynx, and larynx/upper trachea (Figure 3). Cancer of thyroid gland, salivary glands, sinuses, muscles, or nerves in the head and neck region are also associated with these diseases [87]. However, these types of cancer are rare than squamous cell carcinomas [88].

### 4.1. Interplay between Tumor Heterogeneity, Exosomes, and EMT in HNC

Tumor heterogeneity signifies the presence of different cell subpopulations within a tumor with distinct molecular and phenotypic characteristics. Numerous genetic mutations, epigenetic alterations, and environmental factors can result in these subpopulations, which have different growth rates, invasion patterns, and treatment responses [89]. EMT contributes to this heterogeneity by producing cells with various phenotypes, such as CSCs and drug-resistant cells [90]. Exosomes contribute significantly to tumor heterogeneity and EMT. For instance, exosomes derived from diverse subpopulations of cells within a tumor can have discrete molecular profiles, representing tumor heterogeneity. These exosomes can also transfer their molecular cargo to recipient cells, thus affecting their phenotype and function [91]. Additionally, exosomes released by EMT cells can induce EMT in recipients by transferring factors that induce EMT, such as TGF-β and SNAIL. By promoting angiogenesis and remodeling the ECM, these exosomes can also facilitate tumor invasion and metastasis. Moreover, exosomes play a role in tumor heterogeneity and progression by suppressing antitumor immune responses and promoting immune evasion [92]. Tumor heterogeneity plays a crucial role in the evolution of HNC and possible resistance to therapy. Tumors having diverse cell populations are more likely to survive and metastasize. EMT has been shown to contribute to tumor heterogeneity and metastasis, permitting cancer cells to invade and migrate. Exosomes have been found to promote EMT in HNC by transferring EMT-related proteins and miRNAs among cells [93]. According to Wang et al. [94], HNC cell-derived exosomes can induce EMT in recipient cells, enhancing invasion and migration. They also found that exosome-mediated transfer of the EMT-inducing protein S100A4 was crucial for this process.

Overall, the interplay between tumor heterogeneity, exosomes, and EMT in HNC is complex and multifaceted. A better understanding of the molecular mechanisms behind these processes could lead to improvements in the outcomes for HNC.

### 4.2. Exosomes and eccDNA in Head and Neck Carcinoma

There is rising interest in the role of extrachromosomal circular DNA (eccDNA) in current oncology research [95]. eccDNA is a circular DNA that exists outside chromosomes and has also been found in exosomes, where its presence suggests that it may contribute to the intercellular transfer of genetic material in cancer [96]. It has also been found that eccDNA is frequently amplified in cancer cells and contributes to tumor heterogeneity and drug resistance [97]. Several studies have reported the detection of cancer-specific mutations and aberrations in exosomal DNA, indicating the potential of exosomal DNA as a non-invasive biomarker for cancer diagnosis and monitoring [98,99]. The genome-wide presence, the differential expression, and the possible mechanisms of eccDNAs have been demonstrated in a study conducted by Sun et al. [100] in esophageal squamous cell carcinomas. Lin et al. [101] investigated the role and mechanism of eccDNA in the cisplatin (DDP) resistance of hypopharyngeal squamous cell carcinoma (HSCC) using subjects in the HSCC cell line FaDu and the DDP-resistant cell line FaDu/DDP. They found some highly expressed genes totally or partially transcribed from eccDNAs-related sequences and confirmed that the encoding gene RAB3B might be amplified from eccDNA [chr1^circle 46219−52682kb^]. Additionally, they demonstrated that RAB3B could endorse DDP resistance in HSCC by inducing autophagy and concluded that the eccDNA possibly plays an important role in DDP resistance by amplifying relative genes. Recently, Peng et al. [102] reported eccDNAdb, the first integrated database of eccDNAs, which provides a landscape of eccDNAs in various cancers. The data were derived from 3395 samples from 57 tumor types, including also HNC. The authors found that eccDNAs exist more ubiquitously than expected in human cancers. They promote immense amplification of genes, leading to high gene expression.

In conclusion, these studies suggest that eccDNAs can be present in exosomes and may serve as biomarkers for cancer diagnosis and prognosis. Nevertheless, further study is required to fully understand how exosomes and eccDNA affect cancer progression and their potential therapeutic use.

## 5. Exosomes in Head and Neck Squamous Cell Carcinoma

The vast majority of cancers of the oral cavity, pharynx, and larynx are considered to be HNSCC. It accounts for 6% of all cancer cases worldwide. Oropharyngeal and laryngeal squamous cell carcinomas are the most common among HNSCCs [103]. It is caused due to alcohol abuse, smoking, and HPV infection [104,105]. Various oncogenic HPVs, primarily HPV-16 and, to a lesser extent, HPV-18, have been observed to cause oropharyngeal cancer [106]. However, vaccination schemes can reduce HPV-positive HNSCC cases, but HPV-negative HNSCC is still a prime concern.

HNSCC cell molecular markers CD44, CD133, and ALDH1 are of high prognostic importance. Hyaluronic acid and MMPs, critical for intercellular interaction and migration, are the ligands for cell surface receptor CD44, which increases the self-renewal property and metastasis of cells [107,108,109]. CD133, a transmembrane protein, is also upregulated [110]. The intracellular enzyme ALDH1 is essential for converting retinol to retinoic acid and plays a part in cellular detoxification [107]. This protein is upregulated in HNSCC cells. Exosomes from different subsets, such as CD3- from CD3+ TDEs harboring immunosuppressive proteins, are present in the serum of HNSCC patients [111].

These subsets of exosomes reprogram the immune cells and act as surrogates of immune suppression in HNSCC. The miRNA profile also represents the molecular basis of the pathogenesis of HNSCC. OSCC cells show increased secretion of exosomal heat shock protein 90 (HSP90), CD9, and epithelial cellular adhesion molecule (EpCAM) that leads to lymph node metastasis and HSP90α and HSP90β double knockdown clearly reduces the number of cancer cells [112]. In HNSCC patients, HIF-1α and HIF-2α are found to promote the production of miR-21 in exosomes, which induces cell metastasis and invasion of OSCC [113,114]. HIF1α also upregulates the expression of vimentin, SNAIL, and TWIST.

The cells acquire the ability to separate from the basement membrane and related ECM components, in addition to developing metastatic capabilities through EMT. Normal cells undergo apoptosis when detached from ECM, a process called anoikis [82]. However, tumors suppress this process due to the presence of bioactive molecules present in TME. Some growth factors and cytokines, particularly EGF, HGF, and IL-6, activate RAS–MAPK, PI3K–AKT–mTOR, and STAT3 pathways [115,116].

## 6. Exosomes in Nasopharyngeal Cancer

Nasopharyngeal cancer (NPC) is a malignancy of nasopharyngeal epithelial cells generally seen at the pharyngeal recess (fossa of Rosenmüller). It is more prevalent in East Africa and Asia, especially in southern China [117]. NPC EBV-associated malignancy is more metastatic than other HNCs, perhaps due to its link viruses. Nasopharyngeal cancer-derived exosomes identified in circulating blood contribute to tumor cell proliferation, angiogenesis, and immune suppression through remodeling TME and induce EMT, thus promoting tumor metastasis and chemoradioresistance [118]. Many kinds of exosomes have been isolated from the plasma of NPC patients. These can be EBV-related exosomes or pure nasopharyngeal carcinoma-derived exosomes, human EBV-transformed lymphoblastoid cell line (LCL)-derived exosomes, mesenchymal stem cell (MSC)-derived exosomes, etc. It can also contain many exosomes derived from dendritic cells and bone marrow progenitor cells, etc. [119,120,121,122,123]. The exosomes of NPC contain tissue-specific proteins such as latent membrane protein 1 (LMP1), an EB viral protein of which CD9, CD63, CD81, and CD82 are common in other exosomes [124]. These biomarkers are helpful in the diagnosis of this disease.

In NPC, the transmembrane region 1 and N-terminus of LMP1 are sufficient to select EVs effectively and are further downstream regulated by interaction with CD63, a conserved tetraspanin-rich protein in late endosomes and lysosomes [125]. Hence, LMP1 always co-purifies with CD63, and CD63-positive exosomes are also enriched in LMP1 [126]. Another protein, transmembrane domains 1 through 4 (TM5-6), colocalizes with CD63 [125]. One study showed that mutants lacking the N-terminus and TM5-6 could not be packaged into EVs and were highly colocalized with endoplasmic reticulum and early endosome markers [125].

Exosomes in NPC patients’ plasma contain a wide range of biological contents, such as ncRNAs and proteins. For example, exosomes containing miR-17-5p aggravate or oppress NPC in the different stages of NPC development and progression. Exosomal miR-17-5p targets BAMBI and regulates the AKT/VEGF-A signaling, promoting neoangiogenesis [127]. NPC cell proliferation and invasion are promoted by another ncRNA, miR-301a-3p, which represses BTG1 [128]. EBV-negative epithelial cell line expressing EGF receptors has been shown to be induced by exosomal LMP1 that affects the TME [129]. For example, this EBV oncoprotein LMP1 induces EMT through TWIST. It is a master transcription regulator in embryogenesis and has a role in metastasis, making NPC highly metastatic in nature [97]. E-cadherin, the primary component of adherent junctions, is downregulated by LMP1 through the SNAIL transcription factor, promoting EMT [130,131].

## 7. Exosomes in Thyroid Cancer

Thyroid cancer (TC) has become the most prevalent and fastest-growing cancer of the endocrine system in recent years [132]. Differentiated thyroid cancer (DTC) accounts for more than 90% of TC cases diagnosed yearly. DTC includes follicular thyroid cancer (FTC), papillary thyroid cancer (PTC), and other subsets of TC, such as anaplastic thyroid cancer (ATC) and medullary thyroid cancer (MTC), which is associated with a poor prognosis [133,134]. Most thyroid cases show a good prognosis with long-term survival rates, but the death risk from recurrent or persistent diseases is significantly high [135].

EMT and CSCs interplay in thyroid cancers, making them highly progressive and invasive. EMT regulatory networks in thyroid malignancy consist of transcriptional control by the TFs, for example, ZEB, SNAIL, TWIST, SOX9, Runx2, Forkhead box D3 (FOXD3), and epigenetic mechanisms, such as microRNA, DNA methylation, and lncRNA functions [136]. The E-cadherin gene (CDH1) is downregulated in the thyroid gland by unusual protein processing, such as hypermethylation of CDH1 promoter and inhibitory effects of Zeb1/2, SNAIL, E12/E47, and TWIST [137,138]. EMT-inducing molecule circPVT1 promotes MTC growth by targeting miR-455-5p to activate the signaling cascade of CXCL12/CXCR4 [139]. Thyroid CSCs transfer the exosomal lncRNA (MALAT1, lncRNA ROR) and the EMT marker SLUG to induce EMT in normal thyroid cells [40]. PTC-CSCs can also transmit the exosomal lncRNA DOCK9-AS2 to PTC, which activates the Wnt/β-catenin pathway for EMT [140]. Overexpression of certain exosomal proteins in PTC patients with lymph node metastasis (LNM), such as SRC, TLN1, ITGB2, and CAPNS1, significantly induces metastasis through EMT [141]. Undifferentiated thyroid cancer also exhibits hypermethylation of thyroid differentiation genes such as SLC5A5 (NIS) and NKX2-1 [142]. Exosomal miR-145 overexpression in TPC-1 cells has been shown to inhibit VEGF secretion and N-cadherin expression, a hallmark of the EMT process, and promote TC growth and metastasis [143]. The cytoskeleton is also reorganized in EMT, giving rise to better cell–matrix contacts. This encourages cell separation from neighboring cells and initiates invasion and migration. Overexpression of vimentin, an intermediate filaments component, is associated with the induction of EMT with elevated mesenchymal markers (N-cadherin, SNAIL, Zeb1, and Slug) in several thyroid cancer cell lines and tissues [144]. Periostin and fibronectin1, another ECM protein, are induced during EMT and alter the ECM composition resulting in invasiveness.

## 8. Exosomes in Salivary Gland Cancer

Other HNC includes salivary gland malignancies, such as mucoepidermoid carcinoma, adenocarcinoma, and adenoid cystic carcinoma (ACC). Salivary gland cancers are uncommon and account for only 3% of HNCs [145]. ACC is the most common among salivary gland carcinomas, with three distinct growth patterns identified in patients, i.e., cribriform, tubular type, and solid type ACC. Due to limited preclinical models and the limited knowledge of EMT-related proteins, formalin-fixed paraffin-embedded tissue samples are used. Cadherin-4 gene (CDH4) codes for non-epithelial R-cadherin and reduced expression of CDH4 mRNA in salivary adenoid cystic carcinoma (SACC) cell lines show a correlation with the invasion, growth, and mobility of SACC cells. The suppression of CDH4 results in the downregulation of E-cadherin, coded by the CDH1 gene [146]. The expression of EMT-related proteins, SNAIL2 and E-cadherin, is negatively regulated in SACC patients (high SNAIL2 and low E-cadherin) and is significantly correlated with the perineural invasion [146]. Studies have confirmed that bioactive substances in salivary exosomes are involved in the progression and metastasis of salivary gland cancer. More specifically, ACC-derived exosomes enhance the invasion and metastasis of ACC cells. ACC-83-derived exosomes promote cell migration and invasion by targeting ZO-1 and β-catenin [147]. Epiregulin-enriched exosomes derived from epiregulin-overexpressing ACC cells enhance the expression of vascular endothelial growth factor (VEGF) receptor 1 in lung endothelial cells to form the premetastatic niche [148].

## 9. Relationship between Exosomes Biogenesis and Actions with Drug Resistance and Environmental Factors

Surgery, chemotherapy, radiotherapy, immunotherapy, targeted therapy, etc., are some effective methods for treating HNC. However, the resistance of tumors to chemo- and radiotherapy is a serious concern nowadays. In normal cells, exosomes remove unnecessary molecules. However, in cancer cells, this process is hijacked to eliminate chemotherapeutic agents, making them drug-resistant cancer cells by shuttling anti-cancer drugs out of the tumor cell in exosomes [149,150]. Exosomal cargoes such as mRNA, miRNA, and proteins can also facilitate cancer drug resistance in drug-sensitive cancer cells [149,150]. Drug resistance through the EMT-mediated signaling mechanism mainly involves anti-apoptotic pathways and drug efflux pump up-regulation [151].

One example of drug resistance through promoting antiapoptotic pathways is cisplatin-resistant OSCC. The exosomal miR-21 transfers the characteristic of cisplatin resistance in OSCC cells by targeting PTEN and PDCD4 [152]. A common signaling pathway, such as the PI3K/AKT pathway in HNSCC cells that regulates proliferation, invasion, apoptosis, and hypoxia-related protein expression, is associated with radioresistance. The major mechanisms behind it are hypoxia, tumor cell proliferation, and intrinsic radioresistance. Exosomes produced by an EBV infection that includes LMP1 promote the PI3K/AKT pathway, which in turn increases the stemness and chemoradioresistance of NPC [153,154]. LMP1 increases the expression of miR-21 and promotes the resistance of NPC cells to cisplatin-induced apoptosis by repressing programmed cell death protein 4 (PDCD4) and Fas-L (Fas-ligand) [153]. Exosomes linked to EBV also include EBNA1, which inhibits miR-200a and miR-200b, promoting EMT and treatment resistance [155].

Various environmental factors have been shown to modify signaling pathways and cellular stress responses that influence exosome biogenesis, secretion, and composition [156]. These factors include inflammation, oxidative stress, hypoxia, nutrient deprivation, temperature and pH changes, environmental toxins and pollutants, such as tobacco smoke, heavy metals, pesticides, and particulate matter, and also drugs, radiation, or surgery. Exosome secretion can be affected positively or negatively by drugs, depending on the drug, such as chemotherapeutics, non-steroid anti-inflammatory drugs (NSAIDs), glucocorticoids, cholesterol-lowering drugs, and antiviral drugs [157,158,159]. The understanding of how drugs impact exosome biosynthesis may hold great promise for new therapeutic strategies against a variety of diseases. It is worth noticing that several studies in both tumor and normal cells have shown that ionizing radiation increases the release of exosomes and alters exosome-based intercellular communication [160]. Additionally, it has been demonstrated that radiation therapy can change the composition of exosomes secreted from tumor cells. An investigation by Jelonek et al. [161] showed that exosomes released by the irradiated HNSCC FaDu cells showed distinct protein expression profiles. Several of these overexpressed proteins are involved in various cellular processes, which implies that radiation-induced changes in cells are reflected in exosome cargo. Similarly, Mutschelknaus et al. [162] reported that exosomes released by irradiated HNSCC cells have the ability to induce a migratory phenotype in recipient cancer cells, possibly by increasing AKT-signaling via radiation-regulated exosome proteins. They concluded that exosomes are attractive targets to improve radiation therapy strategies because of their potential to drive HNSCC evolution during radiotherapy. There is increasing evidence that exosomes play a key role in radioresistance development. Exosomes, non-coding RNAs, proteins, and the interplay between apoptosis and survival are the main players in exosome-mediated radiosensitivity [163]. Thus, according to a study, exosomes resulting from irradiated HNSCC cells are capable of transmitting pro-survival signals via their exosome charges to recipient cells [164]. Moreover, other researchers have shown that radiation-induced exosomal miR-208a increased the radioresistance and proliferation in lung cancer cells by activating the AKT/mTOR pathway [165]. In contrast, other reports were controversial. In a recent study, Wang et al. showed that autocrine secretions increase radioresistance of H460 NSCLC cells in an exosome-independent way and primarily affect DNA restoration [166]. These studies suggest that radiation-derived exosomes are vital players in cancer radioresistance, mainly through the reprogramming of their charges and facilitating intercellular communication. However, their exact role in this process has yet to be defined and could be affected by factors such as tumor nature and microenvironment, experimental methods, or treatment modalities. Exosomes have also been demonstrated to involve in wound healing and tissue repair after trauma. Following major surgery, immune and stem cells release exosomes as part of the body’s response to tissue damage and inflammation. This could inhibit inflammation, control immune responses, and promote angiogenesis, cell proliferation, and tissue regeneration [167]. Additionally, exosomes facilitate communication between immune cells and also activate and recruit other immune cells to the site of injury or infection. This could be vital in the immediate postoperative period, as the immune response is a key factor in preventing tissue infection and assisting proper wound healing [168]. Conclusively, the exosomes released after trauma or surgery appear to play a significant role in healing. Although the exact mechanism is still unclear, current research aims to identify exosomes as promising targets for future therapeutic interventions [169].

Overall, several environmental factors can have a significant impact on exosomes. Nevertheless, further research is required to fully elucidate how these specific environmental stressors may affect exosome biology.

## 10. Exosome Application in Diagnosis and Therapeutics in HNCs

Exosomes have emerged as promising diagnostic tools due to their aptitude to reflect the molecular content of their parent cells and their potential to be isolated from various bodily fluids, such as blood, urine, and cerebrospinal fluid. Accordingly, exosomes have been identified as potential biomarkers for the diagnosis and monitoring of HNC. Preclinical studies have displayed that those exosomes resulting from HNC cells carry various biomolecules, including microRNAs, DNA, and proteins, which can be used for diagnosis. For example, exosomes derived from oral squamous cell carcinoma cells have been shown to carry specific microRNAs that are differentially expressed in cancer patients in comparison to healthy controls [170]. Notably, exosomes derived from HNC cell lines contain cancer-specific proteins that can be used for early detection and monitoring of the disease [171]. Additionally, exosomes have been shown to play an important role in tumor immunity and angiogenesis in HNC [92,126]. Clinical studies have also demonstrated the potential of exosome-based diagnostics for HNCs. For example, He et al. [172] demonstrated that salivary exosomal miR-24-3p is highly sensitive and specific for detecting OSCC. Similarly, Luan et al. [173] reported that the meta-signature miRNA profiles found in nasopharyngeal carcinoma could be used to develop diagnostic and prognostic biomarkers with clinical utility. Moreover, exosomal proteins and nucleic acids have been found to be useful for monitoring HNC treatment response and predicting disease recurrence [174].

In Table 1, the exosomes and their cargoes as biomarkers for diagnosis or prognosis of HNC are presented. These EVs are associated with the onset and development of HNC and are good candidates for tumor liquid biopsies. CD9, caveolin 1 (CAV1), tumor rejection antigen 1 (gp96), exosomal miRNAs (e.g., miR-486-5p, miR-486-3p, and miR-10b-5p are only secreted by cancer cells) have diagnostic value in HNSCC, and increased expression of HSP90, miR-31, miR-21, and MALAT1 are biomarkers for OSCC. miR-BART7-3p hsa-miR-24-3p, hsa-miR-891a, hsa-miR-1064a-5p, hsa-miR-20a-5p and hsa-miR-1908 help in diagnosis of NPC, while HSP27, HSP60, and HSP90 can act as biomarkers for TC [140,175,176].

Exosomes are more advantageous for the transportation of cargo than liposomes because they may convey contents between cells and shield them from deterioration more efficiently. Cancer treatment can also make use of siRNA that has been exosome-protected (Table 2). Transient receptor potential polycystic 2 (TRPP2) regulates EMT in human laryngeal squamous cell carcinoma to promote invasion and metastasis. Exosomal administration of TRPP2 siRNA, however, inhibited EMT in the FaDu cell line, a cell line of human pharyngeal squamous cell carcinoma, by lowering TRPP2 expression. Vimentin and N-cadherin expression levels were downregulated, whereas E-cadherin expression was elevated by the exosome/TRPP2 siRNA combination [183]. Exosomes can be employed as vectors not just to cure cancer but also to combat drug resistance. It has been recently shown that cetuximab inhibits exosome vesicle synthesis and lowers exosome secretion to cause EMT in OSCC cells [184]. Normal epithelial cells are transformed into mesenchymal phenotypes by OSCC-derived EVs, and cetuximab, an anti-EGFR therapeutic antibody, prevents this cancer-causing action [184].

Collectively, the obtained results regarding the utilization of exosomes in diagnosis and therapeutic targeting are promising. However, further research is needed to improve exosome-based diagnostics for HNC. It encompasses the development of standardized methods for identifying and isolating exosomes as well as validating those methods in various patient populations. Even so, exosome-based diagnostics may lead to improved diagnosis, more personalized treatment, and enhanced patient outcomes for HNC. It turns out that exosomes hold great promise as a non-invasive and reliable diagnostic tool for HNCs.

## 11. Concluding Remarks

In this review, we elaborated upon exosomes, exosome emergence as vehicles for information transfer between cells, their role in HNC progression, and invasion by EMT. Understanding the mechanisms underlying exosome biogenesis, metastasis, and drug resistance at the molecular level aid in designing novel therapeutic targeting in exosome-mediated tumorigenesis metastasis and chemoresistance in HNC. The pathogenesis of HNC is still not fully understood. Exploring exosomes in understanding HNC pathogenesis, diagnosis, and therapeutics can significantly reduce the risk of head and neck cancer spreading to other body parts. The properties of exosomes to deliver bioactive molecules in recipient cells and in TME can be exploited more for an effective cancer treatment strategy which is still a challenging task. Due to the idea that tumor-derived exosomes not only transmit instructions from the tumor to nearby or distant normal cells but also modify critical functional properties and phenotypes as well as activities of these target cells to accelerate tumor growth, they have attracted attention, becoming an emerging area of research. One of the potentials of exosomes is to reverse drug resistance in cells; for example, miRNAs delivered by exosomes can reverse drug resistance in a particular cancer type. In turn, exosome-based therapeutic techniques for cancer therapy, although they have a long way to go, could be a significantly valuable tool for future diagnostic purposes and therapeutical interventions.

## Figures and Tables

**Table 1:** Exosomes with biomarkers for different HNCs.

HNC Type	Biomarker Types	Biomarker Name	References
Papillary Thyroid Cancer	miRNAs	miR-16-2-3p, miR-34c-5p, miR-182-5p, miR-223-3p, miR-223-5p, miR-146b-5p	[177]
Papillary Thyroid Cancer, Follicular Thyroid Cancer	Protein	Thyroglobulin	[178]
Head and Neck Squamous Cell Carcinoma	miRNAs	miR-486-5p, miR-486–3p, miR-10b-5p	[179]
Nasopharyngeal cancer	Protein	EBV, LMP1	[180]
Nasopharyngeal cancer	Protein and miRNA	galectin-9, miR-24-3p	[181,182]

**Table 2:** Exosomes use in therapeutics of HNCs.

HNC Type	Exosome Cargoes	Function	References
Oral squamous cell carcinoma	miR-34a	Tumor suppressor	[185,186,187,188]
Nasopharyngeal cancer	miR-299-3p	Targets MMP-2 to inhibit cell proliferation and migration	[57]
Laryngeal squamous cell carcinoma	TRPP2 siRNA	EMT induction is targeted	[183]
Head and Neck Squamous Cell Carcinoma	let-7	Tumor suppressors; targets the K-RAS oncogene	[189]

## Data Availability

Not applicable.
